# Extravasation of Intravenous Acyclovir in a Patient With Human Herpesvirus 7 Encephalitis: A Case Report

**DOI:** 10.7759/cureus.86242

**Published:** 2025-06-17

**Authors:** Weeratian Tawanwongsri, Sasipaka Sindhusen, Pitchaya Jaruvijitrattana, Thanapon Sutharaphan, Taptim Stavorn

**Affiliations:** 1 Division of Dermatology, Department of Internal Medicine, Walailak University Hospital, Pho Thong, THA; 2 Division of Dermatology, Department of Internal Medicine, Bhumibol Adulyadej Hospital, Bangkok, THA; 3 Division of Dermatology, Department of Internal Medicine, Chao Phraya Abhaibhubejhr Hospital, Tha Ngam, THA; 4 Division of Dermatology, Department of Internal Medicine, Dr. Biew Skin Clinic, Nai Wiang, THA; 5 Division of Dermatology, Department of Internal Medicine, Buriram Hospital, Nai Mueang, THA

**Keywords:** acyclovir, encephalitis, extravasation injury, infusions, tissue necrosis

## Abstract

Acyclovir is a widely used antiviral agent, but intravenous administration can cause cutaneous adverse reactions, particularly extravasation. We report a rare case of acyclovir extravasation in a 20-year-old female patient with human herpesvirus 7 (HHV-7) encephalitis, who developed immediate vesicular skin lesions at the infusion site. The rapid improvement after drug withdrawal and cold compression favored extravasation over herpes infection progression or drug eruption. Acyclovir’s high osmolality and alkalinity contribute to tissue damage. While no standard protocol exists, early detection, infusion discontinuation, aspiration, saline infiltration, cold compression, and hyaluronidase can help minimize injury. This case highlights the importance of prompt recognition and management to prevent complications.

## Introduction

Acyclovir is a nucleoside analog and serves as the first-line therapy for infections caused by herpes simplex virus (HSV) types 1 and 2 (HSV-1 and HSV-2) as well as varicella-zoster virus (VZV) [1]. It selectively targets herpesviruses through activation by the viral thymidine kinase, which phosphorylates it to acycloguanosine monophosphate. Host cellular kinases then convert it to the active triphosphate form.

Acyclovir triphosphate inhibits viral DNA polymerase and is incorporated into viral DNA, causing chain termination and blocking viral DNA synthesis and replication [2]. While generally well tolerated, intravenous (IV) acyclovir can cause cutaneous adverse reactions, particularly when extravasation occurs. Extravasation, the unintended leakage of a drug into surrounding tissues, can lead to chemical inflammation, tissue necrosis, and delayed healing, especially with vesicant or irritant drugs [3]. Despite its clinical significance, acyclovir extravasation remains rare, with limited case reports documenting its presentation and management, and cutaneous drug reactions due to extravasation are estimated to occur in approximately 1.7% of cases [4].

Extravasation refers to the inadvertent leakage of IV medication into the surrounding tissue. Depending on the properties of the infused agent, this can lead to complications ranging from mild irritation to severe tissue injury. For vesicant or irritant drugs such as acyclovir, extravasation may result in chemical inflammation, tissue necrosis, and potential long-term morbidity if not promptly identified and managed. Extravasation injuries are often driven by the osmotic characteristics and pH of the infused solution. Acyclovir has an osmolality of 278 mOsm/kg and a high alkalinity (pH = 11), both of which can induce chemical inflammation and significant tissue damage, ultimately resulting in cell death and necrosis [5,6]. The high osmolality of acyclovir solutions can exacerbate tissue injury by causing osmotic shifts, cellular dehydration, and inflammatory responses. Furthermore, its high alkalinity facilitates the breakdown of proteins, degradation of collagen, and disruption of cellular membranes, enabling hydrogen ions to penetrate deeper tissues and resulting in liquefactive necrosis [7].

Extravasation is a significant clinical concern due to its potential to cause tissue injury; however, it is largely preventable. Early detection and close monitoring during medication administration are key strategies to minimize further tissue damage and improve patient safety. As acyclovir is the drug of choice for patients with suspected or confirmed herpes encephalitis, many of whom may be unable to report early signs of extravasation due to a comatose state. In such cases, the risk of delayed detection and subsequent tissue injury is significantly increased.

## Case presentation

A 20-year-old obese female patient presented to the outpatient clinic with acute fever, headache, malaise, nasal discharge, and nasal congestion. She denied any history of skin rashes. She was discharged home with supportive medications, including paracetamol and cetirizine. Five days after this, she was brought to the emergency department after experiencing a generalized tonic-clonic seizure lasting five minutes, followed by an altered level of consciousness, as well as urinary and fecal incontinence. The patient was admitted to the intensive care unit (ICU). A computed tomography (CT) scan of the brain revealed no evidence of hemorrhage, ischemia, or leptomeningeal enhancement.

Following endotracheal intubation, stabilization of vital signs, and seizure control with phenytoin, levetiracetam, and diazepam, a lumbar puncture was performed. Cerebrospinal fluid (CSF) analysis revealed clear fluid with normal opening pressure, normal glucose and protein concentrations, a white blood cell count of 15 cells/µL composed entirely of mononuclear cells (reference range: <5 cells/µL), and a red blood cell count of 3 cells/µL (reference range: 0 cells/µL). Intravenous acyclovir (10 mg/kg) was initiated, with the dose calculated based on adjusted body weight to optimize efficacy and safety. The drug was diluted in 5% dextrose to achieve a final concentration of 5 mg/mL. CSF Gram stain showed no organisms, and aerobic culture subsequently demonstrated no growth. Polymerase chain reaction (PCR) testing for the meningitis/encephalitis panel later confirmed the diagnosis of HHV-7 encephalitis.

**Figure 1:**
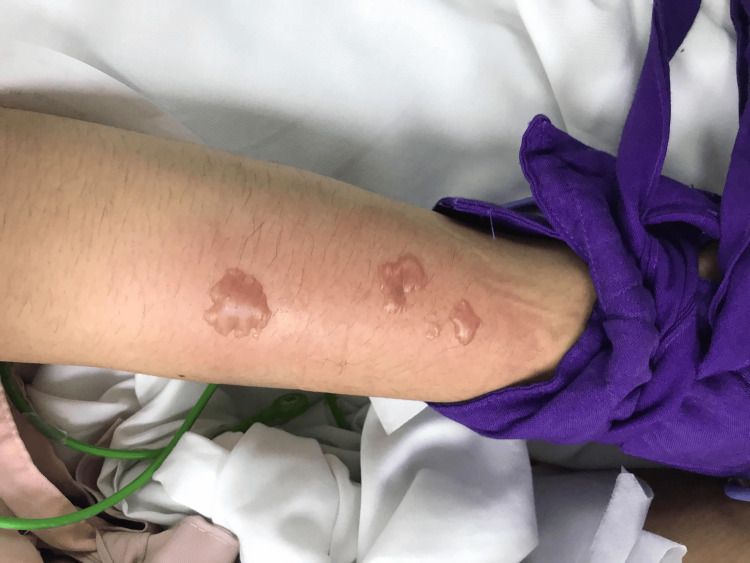
Clinical photograph of the right forearm taken within five minutes after completion of intravenous acyclovir infusion, showing ill-defined erythematous, edematous, and warm plaques with a few small (2–3 cm), yellowish, fluid-filled vesicles.

Acyclovir was administered via a peripheral vein in the left forearm over one hour. Within five minutes after completion of the infusion, skin lesions were observed at the injection site (Figure 1). On examination, the affected area of the left dorsal forearm measured approximately 9 × 4 cm and exhibited ill-defined erythematous, edematous, and warm plaques. Within this area, four discrete, tense, yellowish vesicles were noted, each measuring approximately 2-3 cm in diameter. Cold compression was applied, and the needle was withdrawn. The patient remained in a comatose state due to postictal status; therefore, pain sensation at the lesion site could not be assessed. The immediate appearance of lesions post infusion, and their rapid improvement with cold compression, supported a diagnosis of acyclovir extravasation rather than viral progression or a delayed-type hypersensitivity reaction. Clobetasol cream was prescribed to be applied to the erythematous area twice daily to reduce inflammation.

**Figure 2:**
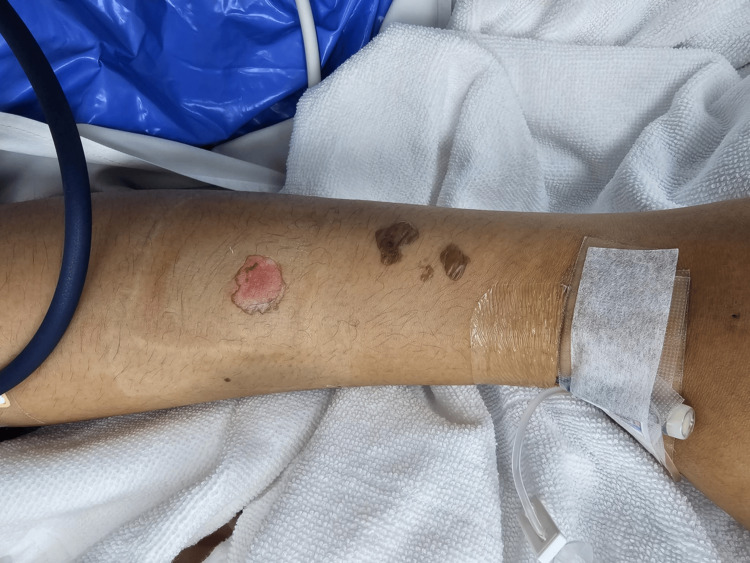
Clinical photograph two days after the initial lesion appearance show that previously noted blebs evolved into shallow erosions, with some becoming flaccid, while erythema and edema resolved.

The second dose was administered via a peripheral vein in the lower leg at the same concentration and infusion rate. Approximately 15 minutes into the infusion, a mild, warm, erythematous, and edematous plaque developed, prompting the discontinuation of the drug. Cold compression was applied immediately, and no overlying blebs were observed. Subsequently, the drug was administered via a peripheral vein in the right forearm with close monitoring. No skin lesions were observed thereafter. On the third day of ICU admission, the lesion on the left forearm was reassessed. The previously noted blebs had evolved into shallow erosions, with some becoming flaccid, while erythema and edema had resolved. A photograph taken two days later (Figure 2) documents these changes.

Additionally, the lesions on the left lower leg had completely returned to a normal skin appearance. The erosion was treated with topical fusidic acid to prevent bacterial colonization. Although histopathological confirmation was not obtained, the diagnosis was based on the temporal association with drug administration and consistent clinical findings, supporting the likelihood of acyclovir extravasation. She regained consciousness, and acyclovir was administered for 14 days to treat encephalitis. She was subsequently extubated safely without additional cutaneous lesions and discharged home. However, during outpatient follow-up, she reported experiencing focal seizures involving the face and limbs, although these had decreased in severity by the two-month follow-up.

At the two-month follow-up, the lesion site had healed completely, with only mild post-inflammatory hyperpigmentation remaining. The patient reported no visible scarring or functional limitations, and no additional vesicles or blebs were observed during this period. Neurological status and antiepileptic therapy were regularly monitored by the neurologist.

## Discussion

This was a rare case of cutaneous adverse reaction due to acyclovir extravasation, manifesting as a vesicular eruption. The differential diagnosis included acyclovir extravasation, herpes infection progression, and drug eruption secondary to acyclovir with recall phenomenon. Key clinical signs of acyclovir extravasation include swelling and induration as early indicators, followed by localized skin discoloration at the infusion site [5]. Patients may also present with pain and erythema, signaling an inflammatory response [3]. Additionally, a lack of blood return from the cannula, though not definitive, is a common finding that may suggest extravasation [8].

The progression of HSV and VZV is a key consideration in the differential diagnosis of vesicular eruptions. HSV infections typically present with multiple small, painful grouped vesicles, often accompanied by fever and lymphadenopathy [9]. VZV infections, particularly disseminated herpes zoster, can similarly manifest as vesicular eruptions, especially in immunocompromised patients. Lau et al. reported a case involving a 55-year-old male patient with HIV who developed herpes encephalitis [10]. The patient presented with diffuse, firm, yet compressible swelling of the right hand, without evidence of skin breakdown or necrosis. Management included wrapping of the affected hand, application of cold compresses, and strict elevation. Similarly, our patient developed skin lesions immediately at the infusion site following drug administration, unlike the delayed onset seen in classic herpes infections. Additionally, the lesions showed rapid improvement after infusion withdrawal and cold compression, further supporting acyclovir extravasation as the more likely cause rather than herpes infection progression. In a drug eruption secondary to acyclovir with recall phenomenon, vesicular lesions may develop. The key characteristic is confluent linear erythema along dermatomes previously affected by a herpes zoster episode, reflecting a hypersensitivity reaction triggered by drug exposure [11]. However, our patient denied any history of skin rashes or prior herpes infection, making this diagnosis less likely in this case.

Several key risk factors contribute to its occurrence, categorized into patient-related, drug-related, procedural, and healthcare provider-related factors. Both very young (younger than six years) and elderly patients (over 65 years) are more vulnerable due to fragile veins and thinner skin [12]. Cancer patients face a higher risk due to repeated venipunctures and compromised venous access from chemotherapy [13]. Neurological, cardiac, and digestive diseases further increase susceptibility [14]. Additionally, patients who are restless or unable to cooperate, such as those with cognitive impairment or severe illness, may inadvertently dislodge IV catheters, increasing the risk of leakage [12]. According to drug-related factors, vesicant and irritant drugs, such as vincristine and doxorubicin, can cause severe tissue damage upon extravasation [15]. Hypotonic or hypertonic solutions can disrupt fluid balance and cause local injury. Similarly, drugs with extreme pH values are more likely to cause chemical irritation and necrosis [7]. According to procedure-related factors, infusion tools and site selection influence extravasation risk. Indwelling and steel needles are linked to higher rates, while infusions near joints are more prone to complications due to movement [12]. Repeated venipunctures weaken veins, increasing the likelihood of leakage [8]. In addition, the experience and training of healthcare providers significantly impact extravasation outcomes. Less experienced nurses may struggle with identifying high-risk veins or recognizing early signs of infiltration. Proper training in IV placement, securement techniques, and early detection can reduce the risk and severity of complications [8]. Our patient was in a comatose state with ongoing seizures, which limited their ability to communicate discomfort or react to early signs of extravasation. This inability to cooperate increased the risk of catheter dislodgement and unrecognized IV infiltration. Additionally, the physicochemical properties of acyclovir contributed to the severity of the reaction.

 The de facto prevalence of acyclovir extravasation-related skin injury remains unclear and may be underestimated in the literature, as such cases are likely underreported despite being encountered in routine clinical practice. Future multicenter cohort studies are needed to better define the incidence, contributing factors, and clinical outcomes of this complication. While there is no universally accepted standard protocol specifically for acyclovir extravasation, general guidelines for managing extravasation injuries can be applied. The most critical step is early detection and immediate discontinuation of the infusion to prevent further leakage and minimize tissue damage [16]. The next step is to aspirate any remaining drug from the cannula without applying pressure to prevent further tissue infiltration. Saline infiltration can be used locally to dilute the extravasated drug and reduce its toxic effects [16]. Cold compresses should be applied to the affected area for 15-20 minutes every four hours during the first 24-48 hours to limit drug dispersion and reduce inflammation [17]. Hyaluronidase, an enzyme that facilitates drug absorption, can be injected subcutaneously around the affected area, particularly for hyperosmolar solutions [5]. Topical corticosteroids may also be applied to reduce inflammation and prevent ulceration [18].

Continuous monitoring is essential to detect worsening symptoms such as increased pain, swelling, or necrosis, with early surgical consultation recommended if symptoms progress. Patient education plays a crucial role in preventing complications, emphasizing the importance of reporting any new symptoms immediately. Staff training is vital to ensure healthcare professionals are well-equipped to prevent, recognize, and manage extravasation effectively [19]. Table 1 outlines suggested steps for managing acyclovir extravasation; these steps should be adapted based on the severity of injury, patient-specific factors, and institutional protocols. In addition, there is a need for close observation of the infusion site during drug administration, particularly in comatose patients, to compensate for their inability to report early symptoms such as pain or discomfort.

**Table 1:** Suggested steps for managing acyclovir extravasation.

Step	Action	Rationale
1. Discontinue infusion	Immediately stop the IV infusion while leaving the cannula in place	Prevents further drug leakage into surrounding tissues
2. Attempt aspiration	Gently aspirate any residual drug through the existing cannula	Reduces the volume of extravasated agent and potential tissue injury
3. Remove cannula	Remove the IV cannula after aspiration	Minimizes additional local trauma
4. Mark and assess the site	Outline the affected area and assess for swelling, erythema, blistering, or induration	Facilitates monitoring of lesion progression and clinical response
5. Apply local cold compression	Apply cold compresses intermittently for 15–20 minutes several times per day (unless warm compresses are preferred based on institutional practice)	Helps reduce inflammation and local tissue reaction; approach may vary depending on drug characteristics
6. Elevate the limb	Elevate the affected extremity	Promotes venous return and reduces edema
7. Close observation	Monitor the site regularly, especially in high-risk patients (e.g., comatose or critically ill individuals)	Enables early detection of evolving complications
8. Specialist referral	Seek consultation with dermatology, surgery, or wound care if signs of necrosis or extensive injury develop	Supports comprehensive management and potential need for debridement or reconstructive care
9. Documentation and education	Document the event thoroughly and inform clinical staff	Enhances patient safety and promotes preventive measures in future practice

## Conclusions

This case highlights the rare occurrence of acyclovir extravasation, which can cause chemical inflammation, tissue necrosis, and delayed healing due to its high osmolality and alkalinity. The patient's immediate skin reaction, rapid improvement after drug withdrawal and cold compression, and absence of herpes infection history support extravasation over herpes progression or drug eruption. While no standard protocol exists, early detection, infusion discontinuation, aspiration, saline infiltration, cold compression, and hyaluronidase are crucial to minimizing tissue damage. Topical corticosteroids, antibiotics, close monitoring, patient education, and staff training further aid in preventing complications. This case underscores the importance of early recognition and prompt intervention to mitigate the adverse effects of acyclovir extravasation.
